# McArdle’s, a Rare Disease That Every Family Doctor Can Manage: A Case Report

**DOI:** 10.7759/cureus.50892

**Published:** 2023-12-21

**Authors:** João R Oliveira, João P Pereira, Mariana F Branco, Luísa C Leite, Ana I Patrício

**Affiliations:** 1 Family Medicine, ACES (Agrupamento de Centros de Saúde) Gondomar, Porto, PRT; 2 Famiy Medicine, SESARAM (Serviço de Saúde da Região Autónoma da Madeira), Funchal, PRT; 3 Family Medicine, SESARAM (Serviço de Saúde da Região Autónoma da Madeira), Funchal, PRT

**Keywords:** fatigue, high carbohydrate diet, exercise intolerance, myalgia, rhabdomyolysis, glycogen storage disorder type v, glycogen, creatine kinase, mcardle

## Abstract

McArdle's disease is a rare autosomal recessive disorder that affects glycogen storage. It typically manifests in adolescence or early adulthood with presenting symptoms, such as fatigue, myalgia, exercise intolerance, and cramps, which can be easily overlooked. This case report seeks to offer a comprehensive overview of the perspective of a patient living with McArdle's disease, emphasizing the importance of treatment encouragement. The report documents the case of a 25-year-old male presenting with myalgia and fatigue exacerbated by strenuous exercise, illustrating the diagnostic challenges associated with McArdle's disease, primarily attributable to clinician unawareness. Furthermore, the case highlights the importance of adhering to lifestyle modifications to mitigate symptoms and prevent flare-ups, as well as the crucial role of the family doctor in such lifestyle maintenance.

## Introduction

McArdle's disease, also known as glycogen storage disease type V, is a rare autosomal recessive disorder typically manifesting in adolescence or early adulthood. The condition is characterized by a deficiency in myophosphorylase enzyme activity, leading to impaired glycogen breakdown within skeletal muscle. This enzymatic insufficiency results in symptomatic manifestations, including exercise intolerance, muscular cramping, and myoglobinuria. Despite limited epidemiological investigations on this disorder, its estimated prevalence in the Spanish population is 1 in 167,000 individuals [1].Therapeutic approaches predominantly center on the adoption of a health-conscious lifestyle. Specifically, adherence to a tailored dietary regimen and engagement in regular, mild-to-moderate physical activities have demonstrated efficacy in ameliorating symptoms and preventing exacerbations. Pharmacological interventions have exhibited marginal effectiveness in this context. The primary objective of this case report is to provide a comprehensive overview of the patient's experience with McArdle's disease, focusing on the clinical presentation, diagnostic process, and subsequent management [2,3].

## Case presentation

The patient was a 25-year-old male employed as a cook, without significant past medical history or regular medication intake. He consumed three to five cigarettes daily and denied substantial alcohol consumption or drug utilization. The patient had a 24-year-old brother being treated in Internal Medicine for polymyositis.

In 2017, at 20 years of age, the patient attended a primary care appointment referring to mild myalgia in the forearms and lower limbs, along with fatigue during moderate physical exertion. There were no relevant findings on physical examination. Blood tests showed no abnormalities, except for a creatine kinase (CK) level of 19,062 U/L (normal range: 55-170 U/L).

Due to the clinical presentation and his brother's medical background, the patient was referred to an Internal Medicine appointment. Electromyography (EMG) testing showed moderate myopathy (Table 1), and autoimmune screening came back negative. He started oral prednisolone (20 mg twice a day) without noticeable clinical improvement after one month. Given the lack of a specific diagnosis at this stage, the patient underwent a deltoid muscle biopsy, which confirmed the diagnosis of McArdle's disease by showing minimal myophosphorylase activity in histochemical staining. Subsequently, the patient's brother also underwent a muscle biopsy, with his diagnosis changing from polymyositis to McArdle's disease.

**Table 1:** EMG patient results EMG – Electromyography; Fib – Fibrillation potential; PSW – Positive sharp wave potential; Amp - Amplitude; Dur – Duration; Poly – Polyphasic motor unit action potentials; Stabil – Stability

Muscle	Spontaneous Activity		Voluntary Activity				Interpretation
	Fib	PSW	Amp	Dur	Poly	Stabil	
Right Posterior Deltoid	0/10	0/10	-	-	Normal	Normal	Myopathy
Left Posterior Deltoid	0/10	0/10	-	-	+	Normal	Myopathy

Corticosteroid therapy was discontinued, and non-pharmacological interventions were recommended such as a carbohydrate-enriched diet, regular light physical activity, and abstaining from tobacco and alcohol.

Over the next two years (2018 to 2020), the patient continued follow-up with both primary care and Internal Medicine. He reported strict adherence to prescribed interventions (dietary plans and regular light exercise) and successfully quit smoking. During this interval, the patient experienced near-complete resolution of all symptoms, only mentioning the need for a longer resting period following more strenuous exercise, with no record of myalgia during normal day routine or exercise.

**Figure 1:**
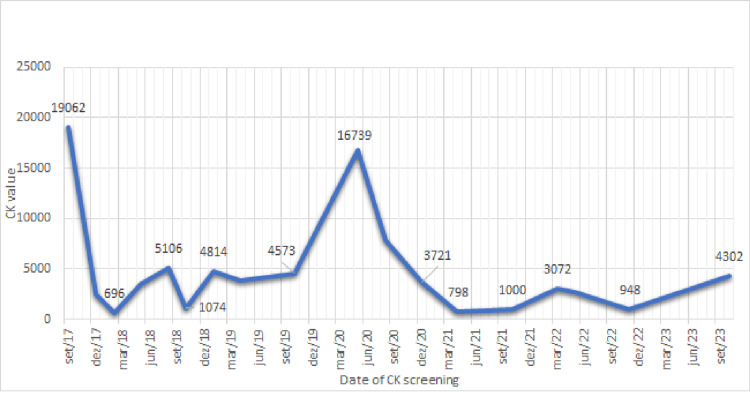
Progression of patient’s CK values in U/L CK - Creatine Kinase; U/L - units per liter

In May 2020, the patient reported a deterioration in symptoms, including generalized myalgia and fatigue with minimal exertion. He mentioned recent non-compliance to the therapeutic regimen and resumed smoking. These behaviors were attributed to job loss and a sense of isolation arising from COVID-19 restrictions. His CK levels rose to 16,739 U/L (Figure 1).

The importance of treatment adherence was then emphasized to the patient. He was also referred for psychological support and smoking cessation sessions, which he followed through.

To date (September 2023), the patient maintains the recommended measures, with CK levels within his baseline range, and remains mostly asymptomatic, only referring to the need for a longer resting period after an occasional physical effort.

## Discussion

McArdle's disease has a high rate of misdiagnosis due to its initial presentation of symptoms such as fatigue, myalgia, exercise intolerance, or cramps [4]. Although common, these symptoms are often undervalued by clinicians. This may lead to significant delays in diagnosis and inadequate therapeutic approaches, as exemplified by the patient’s brothers’ diagnostic journey [5].

The lack of awareness of McArdle's disease and its management can also be demonstrated by the diagnostic method used in this case. While muscle histology is a possible diagnostic approach, it is not the preferred method. Instead, the diagnosis should be achieved non-invasively through a nonischemic forearm exercise test, followed by genetic analysis if results are suggestive of myophosphorylase deficiency [6].

The importance of therapeutic adherence should also be emphasized. Treatment is based on dietary measures and the promotion of a healthy lifestyle, with regular light-moderate exercise and abstinence from both tobacco and alcohol consumption. Higher-carbohydrate diets have shown some improvement in disease progression by maintaining hepatic glycogen levels, which support hepatic glucose mobilization during exercise [7]. Even though exercise can cause muscular breakdown, physical inactivity may worsen exercise intolerance in McArdle’s disease by further reducing the limited oxidative capacity resulting from blocked glycogenolysis [8]. In this case, the relevance of appropriate treatment adherence is made particularly clear by the rapid and marked deterioration in both symptoms and lab results following a short period of non-adherence.

## Conclusions

This case report emphasizes the difficulties in the diagnosis of McArdle’s disease, mostly due to a lack of awareness of the disease and the importance of therapeutic adherence. Further emphasis should be placed on educating clinicians about this condition so that patients like these can receive accurate and prompt diagnosis and treatment.
